# Postoperative Bleeding After Coronary Artery Bypass Grafting

**DOI:** 10.21470/1678-9741-2023-0279

**Published:** 2024-04-15

**Authors:** Mesut Engin, Mustafa Abanoz, Ahmet Kağan AS, Ufuk Aydın, Yusuf Ata, Senol Yavuz

**Affiliations:** 1 Department of Cardiovascular Surgery, University of Health Sciences, Bursa Yuksek Ihtisas Training and Research Hospital, Yildirim, Bursa, Turkey. E-mail: mesut_kvc_cor@hotmail.com; 2 University of Health Sciences, Mehmet Akif Inan Training and Research Hospital, Department of Cardiovascular Surgery, Sanliurfa, Turkey

Dear Editor,

We have read the article by Badem et al.^[1]^ entitled “Plasma Calcium Level and C-Reactive Protein Albumin Ratio Affect Severe Bleeding After Coronary Artery Bypass Grafting” with great interest. First of all, we congratulate the authors for their valuable contribution to the literature. However, we would like to discuss some points about postoperative bleeding.

In that current study, the authors concluded that parameters such as albumin, calcium, and C-reactive protein from preoperative blood values may have an effect on postoperative bleeding^[1]^. All patients underwent coronary artery bypass grafting (CABG) operation accompanied by cardiopulmonary bypass (CPB). Were standard CPB systems used in all patients? Were the pump line lengths the same in the patients? Is albumin added to prime solutions? How did they achieve initial and maintenance diastolic arrest in their patient group? As it is known, different initial cardioplegia solutions may affect clinical outcomes^[2,3]^. Recently, minimally invasive extracorporeal circulation (MiECC) circuits have also been used in CABG^[4]^. Was MiECC used in their patient group? Since all these may affect postoperative bleeding, clarification will increase the value of the study.

Also, the authors stated that “activated coagulation time (ACT) has been measured every hour, and an additional dose of protamine sulfate has been administered when it was > 120 seconds”. In their clinical practice, how much protamine sulfate do they administer to each patient with an ACT of 140 seconds, for example? In addition, the authors stated that arterial blood gas analysis and electrolyte monitoring have been performed every hour for the first six hours. According to these results and bleeding status, were patients given calcium or Transamine® treatment? Has their study group used Haemocomplettan®, cryoprecipitate, and platelet concentrate treatments? In addition, mortality rate in their patient group with drainage > 1000 cc is approximately 13 times higher than the other group. How do they explain this situation? Does bleeding > 1000 cc in just 24 hours explain this?

As they mentioned in the discussion section of the study, CPB circuits affect blood structures^[5]^. The study was planned as a prospective study. For this reason, we think that the absence of postcardiotomy blood parameter evaluation of patients should be added as an important limiting point. Evaluations in the study were made from preoperative blood parameters. As a result, preoperative blood parameters may be affected by CPB systems to varying degrees.

Finally, at the end of their study the authors stated that “As far as we know, our study was the first to determine that low albumin levels in the preoperative period predicted postoperative bleeding”. However, studies conducted by Engelman et al.^[6]^ and Montazerghaem et al.^[7]^ have revealed the relationship between preoperative low albumin levels and postoperative bleeding.
